# Supplementing Genistein for Breeder Hens Alters the Fatty Acid Metabolism and Growth Performance of Offsprings by Epigenetic Modification

**DOI:** 10.1155/2019/9214209

**Published:** 2019-03-26

**Authors:** Zengpeng Lv, Hao Fan, Bochen Song, Guang Li, Dan Liu, Yuming Guo

**Affiliations:** State Key Laboratory of Animal Nutrition, College of Animal Science and Technology, China Agricultural University, 2 Yuanmingyuan West Road, Beijing 100193, China

## Abstract

The experiment was designed to clarify the effect and molecular mechanism of maternal genistein (GEN) on the lipid metabolism and developmental growth of offspring chicks. Laying broiler breeder (LBB) hens were supplemented with 40 mg/kg genistein (GEN), while the control group was fed with the low-soybean meal diet. The offspring chicks were grouped according to the mother generation with 8 replicates each. Hepatic transcriptome data revealed 3915 differentially expressed genes (DEGs, *P* adjusted < 0.05, fold change > 1.5 or fold change < 0.67) between chicks in the two groups. Maternal GEN activated the GH-IGF1-PI3K/Akt signaling pathway, which promoted the developmental processes and cellular amino acid metabolic processes, as well as inhibited the apoptotic process. GEN treatment significantly increased the weight gain, breast muscle percentage, and liver index in chicks. PANTHER clustering analysis suggested that maternal GEN enhanced the antioxidant activity of chicks by the upregulation of gene (SOD3, MT1, and MT4) expression. Accordingly, the activities of T-AOC and T-SOD in the liver were increased after GEN treatment. The overrepresentation tests revealed that maternal GEN influenced the glycolysis, unsaturated fatty acid biosynthesis, acyl-coenzyme A metabolism, lipid transport, and cholesterol metabolism in the chick livers. Hepatic cholesterol and long-chain fatty acid were significantly decreased after GEN treatment. However, the level of arachidonic acid was higher in the livers of the GEN-treated group compared with the CON group. Moreover, GEN treatment enhanced fatty acid *β*-oxidation and upregulated PPAR*δ* expression in the chick liver. ChIP-qPCR analysis indicated that maternal GEN might induce histone H3-K36 trimethylation in the promoter region of PPAR*δ* gene (PPARD) through Iws1, methyltransferases. It also induced histone H4-K12 acetylation at the PPARD promoter through MYST2, which activated the PPAR signaling pathways in the chick livers. In summary, supplementing LBB hens with GEN can alter lipid metabolism in the offspring chicks through epigenetic modification and improve the antioxidative capability as well as growth performance.

## 1. Introduction

Maternal nutrition during gestation has potentially adaptive effects on the phenotype of animal offspring [1]. Programmed metabolic adaptations that occur in utero alter postnatal growth and body composition [2, 3]. Studies identified that placental changes during gestation can induce histone H3 hyperacetylation in the liver of rat offsprings [4]. Epigenetic adaptations occurring during the early developmental period are associated with increased susceptibility for adult-onset metabolic diseases [5, 6]. Thus, epigenetic modifications induced by nutritional changes in the embryo and neonate may influence metabolic status during adulthood. Histone acetylation regulates many cellular processes, but single or multiple acetylation sites cause different effects [7]. Acetylation has been suggested to activate gene transcription, whereas deacetylation mainly represses transcription. Histone methylation is a dynamic change that is strictly regulated by histone methylase and demethylase [8]. This change plays a critical role in transcriptional regulation, genome stability, disease development, developmental growth, and chromatin remodeling, which is gradually erased during the early embryonic period [9, 10]. However, recent studies revealed that histone methylation also be cross-generationally inherited [11, 12]. Therefore, maternal nutrition that affects epigenetic patterning can potentially influence the adult phenotype via alterations in transcriptional promoters.

Genistein (GEN, 4′,5,7-trihydroxyisoflavone), a type of isoflavone (ISF), exists widely in soybean products. It has been used for the treatment of cancer, cardiovascular, and estrogen-dependent diseases [13]. GEN also reportedly activated peroxisome proliferator-activated receptors (PPARs) [14]. PPARs can combine with the retinoid X receptor to form a heterodimer, which combines with PPAR response element in the promoter region [15]. Studies suggest that PPAR*δ* can promote fatty acid catabolism by enhancing fatty acid transport and *β*-oxidation [16]. Accordingly, ISFs reportedly decrease plasma triglyceride (TG) and cholesterol (CHOL) levels in hens fed with high-fat diets along with an increased expression of PPARs [17]. The phenolic hydroxyl in the structure of GEN can bind oxygen-free radicals as hydrogen donors. The antioxidant activity of GEN is stronger than ascorbic acid and quercetin [18]. It is reported that 40 or 80 mg/kg dietary ISF increases the antioxidant capability (T-AOC) and superoxide dismutase (T-SOD) activity of chick plasma [19]. As a DNA methyltransferase inhibitor, ISFs can reduce DNA (cytosine-5)-methyltransferase 3B expression. Recent research indicated that GEN treatment on maternal mice alleviated obesity along with DNA epigenetic modification, and this effect can continue until offspring adulthood [20]. ISFs also reportedly inhibit cervical cancer, prostate cancer, and oesophageal cancer by regulating histone acetylation and DNA methylation [21–23]. Previous studies in our laboratory have indicated that supplementing 40 mg/kg GEN for LBB hens significantly increased hepatic PPAR*δ* expression, which regulates fatty acid metabolism in embryos. Interestingly, the effects of maternal GEN on the metabolism, growth, and antioxidant status of hatched chicks are still unknown. Additionally, its effects on histone modification in progeny are currently rarely reported.

The nutritional supply for mammalian foetuses depends on the maternal metabolism and placental transport [24]. However, the development of chick embryos and newly hatched chicks entirely depends on the nutrients deposited in the eggs. GEN in the body exists mainly in the form of sulfate and glucuronide, and dietary ISF for laying hens reportedly can be deposited in the egg yolk [25]. LBB hens during the late egg-laying period were sensitive to estrogen-like substances, and the phenotypic data of their offspring are plentiful and repeatable. Therefore, we chose birds in two generations as the animal model. In the current research, we used RNA-Seq to study the effects of maternal GEN on the hepatic transcriptome of offspring chicks, aiming to explore the changes in lipid metabolism, growth performance, and antioxidant status. Furthermore, we used the ChIP-qPCR technology to clarify the regulatory mechanism of maternal GEN effect on the key metabolism-related genes in relation to histone modification.

## 2. Materials and Methods

### 2.1. Materials

GEN is a synthetic product from Kai Meng Co. (Xi An, China) Chemical Plant with 99.8% purity.

### 2.2. Animals and Experimental Design

All procedures for animal handling were conducted under protocols approved by the Animal Welfare Committee of China Agricultural University (CAU/NO.160515-2). The experiment was carried out on LBB hens and male offspring chicks at a commercial farm (Zhuozhou, China) under standard conditions. The adaptation period lasts for 2 weeks. Then, 480 57-week-old Ross 308 LBB hens were allocated into two groups (Ab and Bb) with 8 replicates of 30 hens. As shown in Table S1, the corn-soybean meal-cottonseed meal (CSCM) diet was formulated to meet the nutritional requirements of LBB hens according to the Nutrient Requirements of Poultry (NRC, 1994) and previous research [26]. The LGE and HGE groups were fed with the CSCM supplemented with GEN at 40 and 400 mg/kg, respectively. The Ab and Bb groups were fed with CSCM diet supplementing with GEN at 0 and 40 mg/kg, respectively. The formal experiment period lasts for 8 weeks. Each hen was allotted 155 g of feed at 6:00 a.m. every day, with free access to water. Male breeders were caged and given a commercial diet. Eggs (20 eggs each replicate) from 65-week-old LBB hens were incubated under 70–80% humidity and 37.8°C. According to the treatment on the maternal generation, chicks were divided into 2 groups (CON and GEN) with 8 replicates (cages) of 10 birds each. The chicks with free access to water and diet were housed in a standard house, which continued to 3 weeks. All chicks were fed with the corn-soybean meal diet (Table S1), which was formulated based on NRC (1994).

### 2.3. Sample Collection and Chemical Analysis

Body weight gain (BWG), feed intake (FI), and feed conversion ratio (FCR) were determined by all broilers in each replicate. On the 21st day of the broiler-raising experiment, one chick from each cage, with a body weight close to the average, was selected for the wing vein blood collection and slaughter after 8 hours of feed deprivation. Serum was centrifuged at 3000 × g for 10 min and stored at –20°C until further use for biochemical analysis. Liver samples for RNA-Seq and metabolic analysis were collected, which were frozen in liquid nitrogen and kept in a freezer (–80°C) immediately. In addition, one another broiler from each replicate, closing to the average bodyweight, was selected for measurements of carcass traits.

### 2.4. Measurements of Serum Biochemical Indexes

Serum TG, TC, and HDL/LDL (high/low density lipoprotein) levels were assayed using assay kits (Unicel DXC 800, Beckman Coulter, California, America). Serum triiodothyronine (T3), tetraiodothyronine (T4), and growth hormone (GH) levels were measured using commercial double-antibody radioimmunoassay kits purchased from Shanghai Institute of Biological Products. The interassay coefficient of variation was 10%.

### 2.5. Measurement of Antioxidant and Fatty Acid Metabolic Indexes

Liver samples were homogenized with saline to make a 10% homogenate. The malondialdehyde (MDA) levels; T-SOD, CAT, and glutathione peroxidase (GSH-Px) activities; and T-AOC of the 10% homogenate were determined by using a kit (Nanjing Jiancheng Inc., China) according to the manufacturer's protocol. Lipids were extracted according to the method of Bligh and Dyer for further analysis of long-chain fatty acids (LCFAs) [27]. Liver TG and cholesterol levels were examined using commercially available colorimetric diagnostic kits (Nanjing Jiancheng Bioengineering Institute, China). The detection of LCFAs in the liver was conducted referring to the method in our previous research [28].

### 2.6. RNA-Seq Analysis

#### 2.6.1. RNA Isolation, Library Preparation, and Sequencing

Total RNA was extracted and purified from frozen liver tissues using TRIzol (transgene) according to the manufacturer's protocols. Total RNA was then quantified using a nucleic acid/protein quantitative measuring instrument (Bio-Rad). Then, the samples were pooled into one per group at a standardized concentration. Next, four RNA samples from each of the four replicates per treatment were packed in dry ice and sent to Macrogen Millennium Genomics for further library preparation and sequencing. RNA-Seq was performed on an Illumina HiSeq 2500 platform using pair-end sequencing with a read length of 126 bp.

#### 2.6.2. Sequence Read Quality Control

According to the method of quality control in the previous reports, raw reads were assessed using FastQC (version 0.10.1) [29, 30]. Reads with adapter, fuzzy N bases, rRNA, sequences shorter than 20 nt, and low quality with Q<20 were trimmed with fastX clipper (version 0.0.13) [31]. All 126 bp double-end reads of 8 samples from 2 groups were separately aligned to the chicken reference genome (*Gallus gallus* 4.0, version 81, Ensembl), using the spliced mapping algorithm in TopHat2 (version: 2.0.9) [32]. Subsequently, reads were counted by gene using HTseq and *Gallus gallus* genome GTF file (*Gallus gallus* 4.0, version 81, Ensembl). All software runs with the default parameters.

#### 2.6.3. Identification of Differentially Expressed Genes and Gene Ontology Terms

Gene expression intensity was calculated using the reads per kilobase per million (RPKM). Differentially expressed genes (DEGs) were analyzed using Cufflinks software [33]. The DEG model library was fitted to library type and treatment effect. DEGs and *P* values were determined using tests based on negative binomial distribution. All obtained *P* values were adjusted for the false discovery rate (*q*_value) with multiple testing procedures used to control for type I errors [34]. Genes with expression levels with a *P* adjusted < 0.05, fold change > 1.5 or fold change < 0.67 between two samples were defined as DEGs. We functionally classified identified DEGs using the PANTHER classification system (http://www.pantherdb.org/) and Kyoto Encyclopedia of Genes and Genomes (KEGG, https://www.kegg.jp/kegg/) database.

#### 2.6.4. RNA-Seq Confirmation and Measurement of DNA Enriched by qRT-PCR

To confirm the sequencing data, qRT-PCR was performed on 17 randomly selected DEGs using similar methods in the previous report [28]. Total RNA was isolated from tissue samples using the TRIzol reagent (Invitrogen, Carlsbad, CA, USA) according to the manufacturer's protocol. Total RNA was reverse-transcribed to cDNA using PrimeScript RT reagent Kit with gDNA Eraser (TaKaRa) according to the manufacturer's instructions. The one-step real-time RT-PCR was performed using SYBR Premix Ex Taq™ (TaKaRa, Dalian, Liaoning, China) in a real-time PCR machine (ABI7500; Applied Biosystems, Carlsbad, CA, USA) following the manufacturer's guidelines. Primers were designed via Primer Express 3.0.1 software (Applied Biosystems) and are shown in Table S2. GAPDH was used as the housekeeping gene. Relative mRNA expression levels of each target gene were normalized to the control using the 2−ΔΔCT method. The DNA enriched by ChIP was amplified by qPCR. The information of primer at the two sites of the promoter region of gene PPARD was shown in Table S3.

### 2.7. Assay Procedure of Chromatin Immunoprecipitation (ChIP)

We conducted the assay according to the method of Saleh et al. [35]. The whole procedure included chromatin crosslinking with tissue samples, chromatin fragmentation, chromatin immunoprecipitation, reverse crosslinking, and DNA purification. The antibodies used in the assay include anti-acetyl-histone H4 (Lys12) (07-595, Millipore, US), anti-acetyl-histone H4 (Lys 8) (A7258, ABclonal, US), anti-acetyl-histone H3 (Lys 4) trimethylation (04-745, Millipore, US), and anti-acetyl-histone H3 (Lys 36) (ab9050, ABclonal, US) antibodies. The improved method is shown in Supplementary File 2.

### 2.8. Statistical Analysis

The results were expressed as mean ± SD or mean ± SEM (for gene expressions), and differences were considered significant when *P* < 0.05, *P* < 0.10 were set as the trend of difference. Treatment means were compared by independent sample *T*-test with SPSS 11.0 for windows.

## 3. Results

### 3.1. Production Performance of Offspring Chicks

As shown in Table 1, adding GEN into the diet of LBB hens significantly increased the body weight gain of chicks during day 1-21. However, feed intake and the feed gain ratio were not significantly different between the CON and GEN groups. The breast muscle rate and liver index were significantly higher in the GEN group than the CON group (*P* < 0.05), while the abdominal fat percentage of the chick was decreased after GEN supplementation for LBB hens (*P* = 0.094). The GEN treatment made no significantly effects on the heart index of chicks.

### 3.2. Lipid Metabolic Indexes and GEN Content in Offspring Chicks

As shown in Table 2, serum CHOL (*P* < 0.01) and LDL levels of chicks from the GEN group (*P* < 0.05) were decreased compared to the CON group, while the serum HDL level was increased after GEN treatment. The levels of GEN in the livers of 21-day-old chicks were not significantly different between the CON and GEN groups (Table 3). Adding GEN to the diet of LBB hens reduced the LCFA (Table 3, *P* < 0.05), cholesterol (*P* < 0.05), and triglyceride (*P* < 0.063) in the chick livers. Remarkably, GEN treatment decreased the levels of C14:0, C21:0, C20:5n3, and C22:6n3 in the chick livers (*P* < 0.05). Meanwhile, C16:0, C17:0, and C18:1n9c content were presented with a decreased trend after GEN supplementation (*P* < 0.05). Hepatic n-6 family LFCAs (*P* = 0.089), monounsaturated LFCAs (MUFAs, *P* = 0.090), and polyunsaturated LFCAs (PUFAs, *P* = 0.078) of the GEN group tended to reduce compared with the CON group. However, the content of C20:4n6 (arachidonic acid) and C23:0 was significantly higher in the GEN group than in the CON group (*P* < 0.05).

### 3.3. Hepatic Antioxidant Indexes of Chicks

As shown in Table 3, adding GEN to the diet of LBB hens increased T-SOD activity (*P* < 0.05) and decreased MDA content (*P* < 0.05) in the liver of chicks. Hepatic T-AOC of the GEN group was significantly higher than the CON group (*P* < 0.05). The activities of CAT and GSH-Px in the liver of the GEN group were not significantly different from the CON group.

### 3.4. The Hormone Levels in the Chick Serum

As we can see from Table 4, the levels of T3 and T4 in serum were not significantly different between the two groups. The level of GH in the serum of the GEN group was presented with an increasing trend compared with the CON group (*P* = 0.067).

### 3.5. RNA Sequencing (RNA-Seq) Data of the Chick Livers

After analyzing the hepatic RNA-Seq data from 21-day-old chicks, we obtained a total of 642,089,356 clean reads, averaging 80,261,169 clean reads (73,869,122 to 87,132,554) per sample (Table S4). The average Q30 value was 91.26%. The average number of reads from all samples aligned to *Gallus gallus* (assembly Gallus_gallus 5.0, https://www.ncbi.nlm.nih.gov/genome/111?genome_assembly_id) was 260,603 with an average alignment ratio of 83.755%. The number of mapped reads on different regions of the genome is displayed in Figure S1.

### 3.6. Confirmation of the Accuracy of the RNA-Seq Transcriptome Data by Quantitative Real-Time PCR

**Figure 1:**
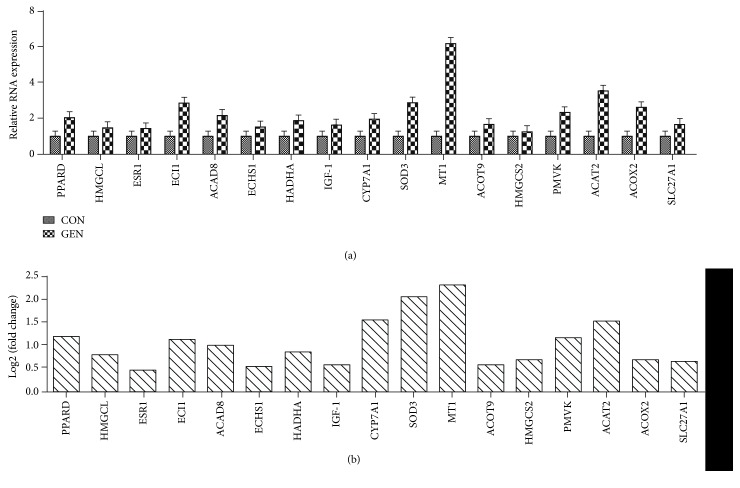
(a) The relative RNA expressions of genes in the chick liver as determined by using qPCR (*n* = 6). (b) The log2 (fold change) of gene expression abundance (GEN vs. CON) as determined by using RNA-Seq (*n* = 4).

To confirm the accuracy of the RNA-Seq transcriptome data, we randomly selected 17 DEGs. The expression levels of selected genes were quantified using qRT-PCR, and the results were consistent with the findings obtained by RNA-Seq (Figures 1(a) and 1(b)). The results suggested that RNA-Seq reliably identified DEGs in the chicken liver transcriptome.

### 3.7. DEG Analysis by Cuffdiff Software and the PANTHER Classification System

**Figure 2:**
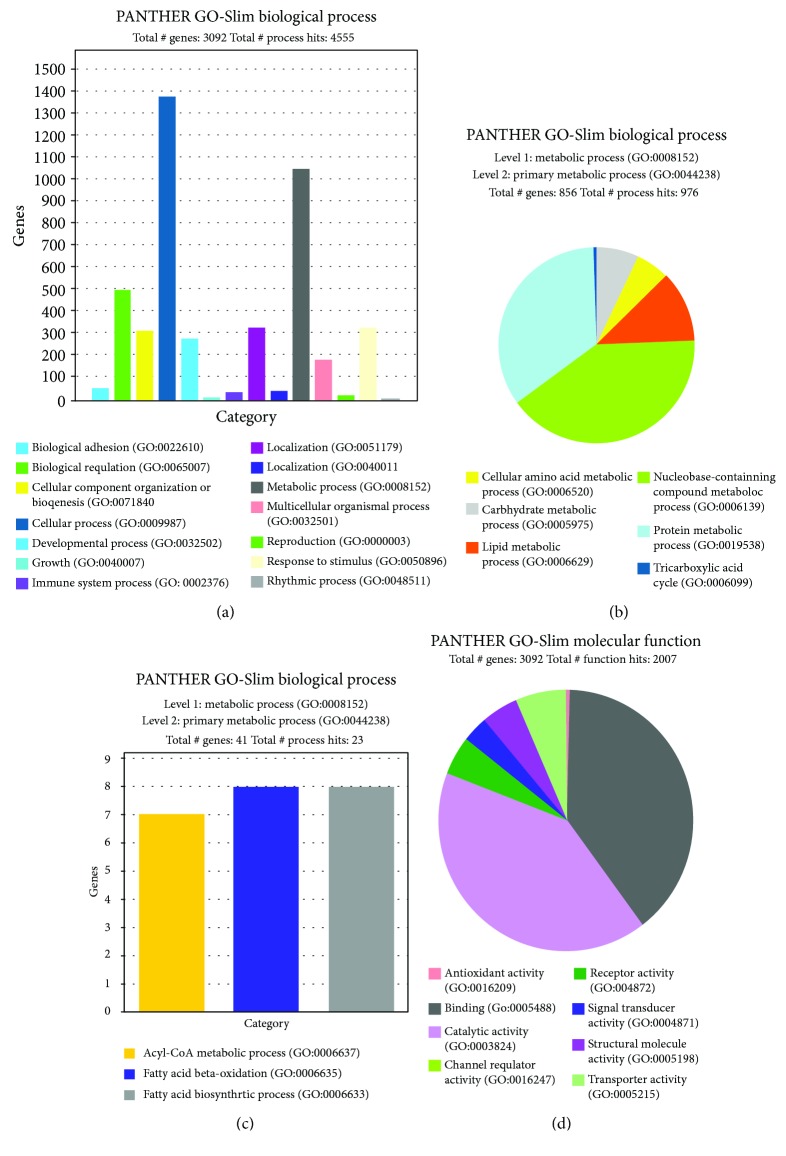
PANTHER GO-Slim analysis. (a) PANTHER GO-Slim analysis of all biological processes. (b) PANTHER GO-Slim analysis of primary metabolic processes. (c) PANTHER GO-Slim analysis of fatty acid metabolism processes. (d) PANTHER GO-Slim analysis of all molecular functions.

As shown in Table S5, 3915 DEGs were obtained between the CON and GEN groups by Cuffdiff software analysis. The expressions and fold change of DEGs discussed in the current paper were shown in Table S6. We further identified 3092 DEGs using PANTHER analysis. As shown in Figure 2(a), 4550 biological processes were obtained by DEG cluster analysis. There were 1377, 1050, 504, 281, and 332 genes, respectively, clustering into the cell process, metabolic process, reproduction, developmental process, and response to stimulus categories. As shown in Figure 2(b), 22 DEGs, including PKM, pyruvate kinase; GANC, glucosidase alpha; RANBP1, Ran-binding protein 1; PGD, 6-phosphogluconate dehydrogenase; TKT, transketolase; PGM5, phosphoglucomutase-like protein 5; HK1/HK2, hexokinase; RPE, ribulose-phosphate 3-epimerase; LDHB, L-lactate dehydrogenase; SYNJ2, synaptojanin 2; PGM2, phosphoglucomutase; HKDC1, hexokinase domain containing 1; RGN, gluconolactonase; and PGM2L1, glucose-1,6-bisphosphate synthase clustered with the monosaccharide metabolic process (GO:0005996). Furthermore, DEGs (LDHB, L-lactate dehydrogenase; CS, citrate synthase; IDH3A, isocitrate dehydrogenase 3 (NAD+) alpha) clustered with the tricarboxylic acid cycle.

We further conducted the adjusted analysis on DEG clusters using Bonferroni statistical overrepresentation tests. As shown in Table 5, 10 DEGs clustered with glycogen metabolism (GO:0005975) and 108 DEGs clustered with lipid metabolism (GO:0006629). In the fatty acid metabolic process (Figure 2(c)), we obtained clustered terms, including the acyl-CoA metabolic process (GO:0006637; ENSGALG 00000013848, mevalonate kinase; ACSM3, medium-chain acyl-CoA synthetase; ACOT9, acyl-CoA thioesterase 9; MVD, mevalonate diphosphate decarboxylase; HMGCS2, hydroxymethylglutaryl-CoA synthase; FAR1, fatty acyl-CoA reductase 1; and PMVK, phosphomevalonate kinase), fatty acid *β*-oxidation (GO:0006635; ACADL, long-chain specific acyl-CoA dehydrogenase; ECHDC1, ethylmalonyl-CoA decarboxylase; CRYL1, Lambda-crystallin homolog; HADHA, trifunctional enzyme subunit alpha; ECI1, enoyl-CoA delta isomerase 1; ETFA, electron transfer flavoprotein subunit alpha; ACOX2, acyl-CoA oxidase; and ACAT2, acetyl-CoA acetyltransferase), and fatty acid biosynthetic process (GO:0006633; PTGES, prostaglandin E synthase; ALOX5AP, arachidonate 5-lipoxygenase-activating protein; and ENSGALG00000011450, leukotriene c4 synthase). The transcriptional levels of the abovementioned genes were significantly upregulated in the livers of GEN-treated LBB hen offspring compared with those of the CON hen offspring (Table S6). In addition, adding GEN into the LBB hen diet significantly promoted offspring lipid transport, cholesterol metabolic processes, and glycolysis (Table 4).

**Figure 3:**
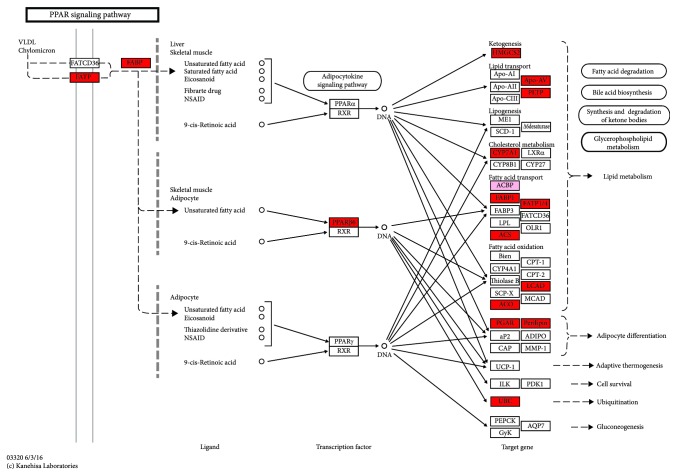
DEGs clustered in the PPAR signaling pathway. Genes marked in red were upregulated (*P* adjusted < 0.05, |fold change| > 1.5). Genes marked in pink were upregulated (*P* adjusted < 0.05, 1.0 < |fold change| < 1.5).

As shown in the KEGG pathway analysis (Figure 3), GEN supplementation for LBB hens significantly upregulated the transcriptional level of VLDL in the liver of offspring and activated the PPAR signaling pathway (PPARD, peroxisome proliferator-activated receptor delta 3.21|7.33; SLC27A1, solute carrier family 27 member 1, 4.96|7.81; ACOX2, acyl-CoA oxidase 2, 80.38|130.25; HMGCS2, hydroxymethylglutaryl-CoA synthase, 567.82|920.71; APOA5, apolipoprotein A-V, 213.7|355.11; PLTP, phospholipid transfer protein, 2.19|5.31; ACSL5, long-chain acyl-CoA synthetase, 96.26|173.67; PLIN1, perilipin-1, 0.67|1.37; ACSBG1, 0.35|0.73; FABP3, fatty acid-binding protein, 2.19|5.69; CYP7A1, cholesterol 7alpha-monooxygenase, 24.28|71.42; ANGPTL4, angiopoietin-like 4, 24.56|93.49; ACADL, long-chain-acyl-CoA dehydrogenase, 339.21|531.46; ACSBG2, acyl-CoA synthetase bubblegum family member 2, 49.74|208.31; VLDL, very low-density lipoprotein, 2.23|4.89; UBE2R2, ubiquitin-conjugating enzyme E2 R2, 12.82|19.80; and DBI, acyl-CoA binding protein, 557.62|721.32). From the overrepresentation test (Table 5), DEGs were clustered into the insulin receptor signaling pathway (IRS2, insulin receptor substrate 2 |0.60; SOCS1, suppressor of cytokine signaling 1 |1.51; PTPN2, tyrosine-protein phosphatase nonreceptor type 2 |1.51; CCND3, G1/S-specific cyclin-D3|1.52; ZNF106, zinc finger protein 106|1.53; AKT1, RAC serine/threonine-protein kinase|1.54; IRS1, insulin receptor substrate 1 |1.56; PHIP, PH-interacting protein |1.56; PRKCD, protein kinase C delta|1.58; NCOA5, nuclear receptor coactivator 5|1.59; PIK3R3, phosphoinositide-3-kinase regulatory subunit 3 |1.67; STXBP4, syntaxin-binding protein 4|1.68; SIK2, serine/threonine-protein kinase SIK2|1.71; TSC1, tuberous sclerosis 1 |1.74; PID1, phosphotyrosine interaction domain containing 1 |1.79; CAV2, caveolin 2 |1.80; NUCKS1, nuclear casein kinase and cyclin-dependent kinase substrate 1 |1.81; SMARCC1, SWI/SNF-related matrix-associated actin-dependent regulator of chromatin subfamily C|1.89; SOGA1, suppressor of glucose autophagy associated 1 |2.01; and PIK3R2, phosphoinositide-3-kinase regulatory subunit 2 |2.09) and insulin-like growth factor receptor signaling pathway (EIF2AK3, eukaryotic translation initiation factor 2-alpha kinase 3 |1.52; AR, aldehyde reductase|1.52; AKT1, serine/threonine-protein kinase|1.54; GIGYF2, GRB10 interacting GYF protein 2|1.54; IRS1, insulin receptor substrate 1 |1.56; PLCB1, phospholipase C beta 1|1.56; IGFBP1, insulin-like growth factor-binding protein 1|1.59; ATXN1, ataxin 1|1.80; CILP, cartilage intermediate layer protein|1.90; and CDH3, cadherin 3|2.20). GEN treatment promoted the cellular amino acid (AA) metabolic process and developmental growth, as well as inhibited the apoptotic process. Among these genes, IGF1 and IGFBP1 expressions were significantly upregulated. Additionally, GEN supplementation activated the MAPK signaling pathway, I-kappaB kinase/NF-kappaB cascade, and response to interferon-gamma in offspring.

Regarding molecular function (Figure 2(d)), DEGs were mainly clustered with catalytic activity (GO:0003824) and binding (GO:0005488). All genes clustered with antioxidant activity (GO: 0016209; MGST3, microsomal glutathione S-transferase 3; MGST2, microsomal glutathione S-transferase 2; SOD3, superoxide dismutase 3; PER2, period circadian regulator 2; PXDN, peroxidase; ALOX5AP, arachidonate 5-lipoxygenase-activating protein; LTC4SL, leukotriene C4 synthase; MT1, metallothionein 1; and MT4, metallothionein 4).

### 3.8. Histone Modification Profile Using RNA-Seq Analysis

The effect of maternal nutrition on the development and metabolism of offspring is regulated by epigenetic modification. Thus, we conducted GO analysis on histone modification using genes that were differentially expressed between the CON and GEN groups. As we can see from Table S7, adding GEN into the diet of LBB hens significantly influenced methylation (GO:0032259) and histone lysine methylation (GO:0034968) in the offspring chick livers. GEN treatment altered histone H3-K36 methylation, histone H3-K36 trimethylation, and histone H3-K36 demethylation and upregulated the expression of specific methyltransferases (SETD2, histone-lysine N-methyltransferase; IWS1, transcription factor SPN1; and NSD1, nuclear receptor binding SET domain protein 1). Additionally, DEGs were clustered into histone H3-K4 trimethylation and histone lysine demethylation. GEN supplementation upregulated histone acetyltransferase (EP300, E1A binding protein p300; EP400, E1A binding protein p400; and MYST2, lysine acetyltransferase 7) expression and promoted histone acetylation, including histone H4-K8 and histone H4-K12 acetylation in chick liver. GEN supplementation for LBB hens also promoted mRNA transcription from the RNA polymerase II promoter and histone monoubiquitination.

### 3.9. Maternal GEN Supplementation Affects Histone Trimethylation and Acetylation at the Promoter Region of PPARD in the Liver of Offspring Chicks

#### 3.9.1. Chromatin Fragmentation Result

Under electrophoretic conditions (2% agarose gel electrophoresis; 140 V; 25 min), most chromatin fragments are between 100 and 500 bp (Figure S3).

#### 3.9.2. ChIP-qPCR Primer Information

We chose two promotor sites on the PPAR*δ* gene (PPARD; chr26:4048020+4048156 and chr26:4047862+4047963) to detect histone modification status. Primer information is shown in Table S3.

#### 3.9.3. ChIP-qPCR Results

**Figure 4:**
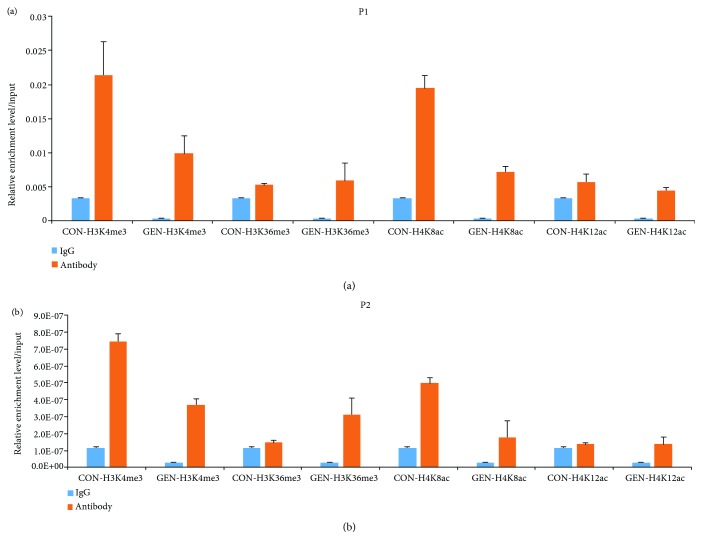
Relative enrichment level/input of qPCR products using the promoter site primer. IgG is the negative control; the four histone modification sites included histone H3 lysine 4 trimethylation (H3K4me3), histone H3 lysine 36 trimethylation (H3K36me3), histone H4 lysine 8 acetylation (H4K8ac), and histone H4 lysine 12 (H4K12ac). The results are expressed as the means ± SD. (a) P1 site (chr26:4048020+4048156); (b) P2 site (chr26:4047862+4047963).

As shown in Figure 4, two primers for the promoter of PPARD gene were used to amplify the DNA enriched by ChIP. We compared DNA enriched by the four antibodies (H3K4me3, H3K36me3, H4K8ace, and H4K12ace) with that by IgG. The results showed the relative amount of enriched DNA precipitated by H3K36me3 and H4K12ac at the P1 and P2 site of the PPARD promoter from the liver of the GEN group was higher than the CON group. However, the relative amount of enriched DNA precipitated by H3K4me3 and H4K8ace at both the P1 and P2 site of PPARD was not significantly different between the two groups. GEN supplementation for LBB hens induced H3K36me3 and H4K12ac modifications at the promoter (chr26:4048020+4048156 and chr26:4047862+4047963) of PPARD in the liver of offspring chicks.

## 4. Discussion

### 4.1. Effect of Maternal GEN Supplementation on the Growth Performance of Offspring Chicks

ISFs reportedly promote the growth and reproductive performance of livestock [36, 37]. It is suggested that dietary GEN 20 to 80 mg/kg not only improves growth performance but also beneficially affects immunological responses in broiler chicks [38]. Transgenerational effects involve epigenetic changes and phenotypic changes induced by maternal and environmental factors, leading to developmental changes in offspring [39]. However, the effect of dietary GEN supplementation for LLB hens on the growth of offspring is still unknown. In this experiment, LLB hens during the late egg-laying period were selected as an experimental model, in which egg-laying rate and egg quality decreased rapidly. Adding 40 mg/kg GEN to the diet of LBB hens significantly increased the body weight gain of chicks during the early growth stage, which is consistent with the increased chicken embryo body size parameters that we previously reported (Figure S2). Protein metabolism in broiler chicks is more robust than fat metabolism during the early growth period. We found that DEGs from the liver transcriptome were clustered into the developmental process and cellular AA metabolic process. Meanwhile, GEN treatment upregulated the insulin signaling pathway and glycolytic process in the chick livers, which promote glucose transformation into AAs.

GEN can reportedly increase the plasma GH concentration in ewes during the infusion period [40]. In the present study, supplementing LBB hens with 40 mg/kg GEN increased the serum GH level in chicks, following with the increased IGF-1 and IGFBP1 mRNA expressions in the liver. The liver can produce IGFs under the action of GH. IGFs have potent mitogenic effects on target organs through receptor- and IGFBP-mediated regulation [41]. Additionally, IGFs can promote DNA synthesis, proliferation, and differentiation, as well as inhibit the apoptotic process [42]. Accordingly, PANTHER overrepresentation tests revealed that GEN treatment inhibited the apoptotic process in chicks. IGF-1 reportedly promotes postnatal development and cell proliferation by activating the PI3K/Akt signaling pathway [43–45]. In our study, maternal GEN supplementation activated the IGFs signaling pathway in the chick livers, with increased PI3K (PIK3CD, PIK3R5) and AKT (AKT1, AKT2) mRNA expression. In summary, we describe here for the first time that adding GEN into the diet of LBB hens can promote the developmental growth of offspring chicks by activating the GH-IGF1-PI3K/Akt signaling pathway.

### 4.2. Effect of Maternal GEN on the Antioxidative Capability of Chicks

Broilers are a type of poultry with a high growth speed and strong metabolic rate, which produces a large number of free radicals. Superoxide anion radical (O2-) induces lipid peroxidation, which can damage the structure and function of biofilms, leading to metabolic disorders in the body [46]. It is reported that ISFs can inhibit the oxidation of active ingredients in the body by binding to specific LDL sites [47]. Therefore, studying the effect of maternal GEN on the antioxidant capacity of offspring is greatly meaningful. In the current study, adding GEN into the diet of LBB hens significantly improved T-AOC and inhibited lipid peroxidation in the chick livers. A SOD-CAT-GSH-Px-based enzyme system in the body plays an important role in the antioxidant processes. Maternal GEN supplementation significantly increased T-SOD activity in the chick livers. PANTHER cluster analysis also showed that GEN treatment affected peroxidase activity (GO:0004601) in the chick liver with upregulated MnSOD (SOD3) mRNA expression. GEN reportedly exerts antioxidant capability by binding to ESRs, leading to the rapid activation of the ERK1/2 and NF*κ*B signaling pathways and a delayed upregulation of MnSOD gene expression in MCF-7 cells [48]. Similarly, maternal GEN supplementation upregulated the transcriptional level of ESR1 in the liver of chicks and activated the I-kappaB kinase/NF-kappaB cascade. Therefore, maternal GEN supplementation might upregulate MnSOD mRNA expression by activating ESR-NF*κ*B in the offspring. Metallothionein has approximately 10,000 times the ROS-scavenging capacity of SOD [49]. In the present experiment, supplementing LBB hens with GEN significantly increased MT1 and MT4 mRNA expression in the chick livers. This finding is consistent with the report that GEN can increase metallothionein expression in Caco-2 cells [50]. Our study provides the first evidence that maternal GEN supplementation can increase SOD and MT mRNA expression, as well as the antioxidant capacity of chicks. Therefore, the effect of maternal GEN supplementation might protect next-generation chicks against oxidative stress, which can improve the growth performance.

### 4.3. Effect of Maternal GEN Supplementation Alters Lipid Metabolism and Enzyme Transcription in Broiler Chicks

Antenatal phytoestrogen exposure can reduce the risk of metabolic syndrome in offspring [51, 52]. GEN supplementation increased VLDL transcription in chicks. The main function of VLDL is to transport hepatic TGs to peripheral tissues, which can decrease the level of TGs in the serum and liver. Furthermore, cluster analysis indicated that GEN treatment affected fatty acid biosynthesis, acyl-CoA metabolic process, and lipid transport in chicks. Further detection showed that GEN supplementation significantly decreased LCFA levels in the liver. GEN reportedly increases energy expenditure and fatty acid *β*-oxidation in hepatocytes [53]. Cuffdiff analysis clearly showed that maternal GEN supplementation significantly upregulated the transcriptional levels of *β*-oxidation key enzymes, including ACADL, ACADSB, ACAD8 (ester acyl-CoA dehydrogenase), HADHA (hydroxyacyl-CoA dehydrogenase), ECHDC (ethylmalonyl-CoA decarboxylase), ETFA (electron transfer flavoprotein subunit alpha), ACOX2 (acyl-CoA oxidase), ACAT2 (acetoacetyl-CoA thiolase), and ACOT (acetyl-CoA C-acetyltransferase). Therefore, GEN supplementation decreased the LCSFA and PUFA levels in the chick livers. ACAT catalyses acyl-CoA hydrolysis into free fatty acid (FFA) and CoA, which promotes fatty acid *β*-oxidation and carboxylic acid transport [54, 55]. Meanwhile, we found that GEN supplementation significantly increased the transcriptional levels of arachidonate 5-lipoxygenase-activating protein, C-fos and Egr-1, as well as the AA levels in the chick livers. Studies have shown that AAs can upregulate the expression of C-fos and Egr-1, which promote cell growth and organ development [56]. This regulation might be one of the pathways by which maternal GEN supplementation promotes progeny growth.

### 4.4. Effects of Dietary GEN Supplementation for LLB Hens on the Cholesterol Metabolism in Offspring Chicks

Fatty acids in hepatic mitochondria are decomposed into acetyl-CoA, which then are mainly converted into energy, cholesterol, ketone bodies, and new fatty acids. Adding GEN into the diet of LBB hens significantly upregulated the transcriptional levels of HMGCoA lyase (HMGCL), HMGCoA synthase (HMGCS2), and 3-oxoacid CoA-transferase (OXCT1) in the chick liver, which promoted ketone body formation. GEN treatment upregulated progesterone kinase (MVD) and phosphomevalonate kinase (PMVK) mRNA expression, which are the key enzymes in cholesterol synthesis. In addition, the cholesterol 7*α*-hydroxylase (CYP7A1) transcription level in the liver and the serum HDL level of chicks were increased after GEN supplementation in LBB hens, but the serum LDL level was decreased. It is well-established that LDL carries cholesterol from the liver to other tissues; HDL sends cholesterol back to the liver; then, cholesterol is metabolized into bile acids or drained from the intestine [57]. CYP7A1 is a key enzyme that catalyses bile acid synthesis from cholesterol. Thus, GEN supplementation for LBB hens promoted the conversion of cholesterol into bile acid in chicks. Accordingly, we found that cholesterols in the liver and serum of chicks were both reduced after GEN treatment. Studies demonstrate that GEN can increase the serum HDLC level and decrease the cholesterol and LDLC level in hyperlipidaemic patients [58], which need further studies to explore the mechanism. It was reasonable to conclude that maternal GEN supplementation promoted cholesterol transportation and decomposition in offspring chicks.

### 4.5. Effects of Dietary GEN Supplementation for LBB Hens on Hepatic PPAR*δ* Expression of Offspring Chicks and Its Mechanism

PPARs play important roles in the regulation of fatty acid metabolism. GEN can promote fatty acid catabolism by activating PPAR*α* and PPAR*γ* [59]. In this study, GEN treatment did not significantly affect PPAR*α* or PPAR*γ* expression in offspring livers but upregulated the expression of PPAR*δ* and target genes. PPAR*δ* is mainly involved in the cellular lipid metabolism, which promotes reverse cholesterol transport and oxidative decomposition of lipid [60]. Furthermore, ACOT, which is strongly activated by PPARs, is able to terminate fatty acid synthesis [61]. Therefore, we speculated that maternal GEN supplementation has the ability to regulate the network that decreases lipid levels in offspring through PPAR*δ*. We further explored the mechanism by which maternal GEN affects PPAR*δ* expression in offspring. First, we doubt that the dietary GEN supplementing for LBB hens can deposit into the chick liver, which serve as the ligand to activate the PPAR*δ* signaling pathway directly. However, the contents of GEN in the chick livers were not different between the CON and GEN group. Interestingly, studies have shown that prostaglandins, eicosanoids, and retinoic acid are natural ligands of PPAR*δ* [62]. Our study showed that hepatic AA levels are significantly higher in GEN-treated offspring chicks compared with the CON group, which might activate the PPAR*δ* signaling pathway.

Epigenetic regulations, including DNA methylation, histone modification, chromatin remodeling, and RNA interference, can be inherited from breeders to offspring, which affect the offspring's development [63, 64]. Cluster analysis indicated that GEN supplementation for LLB hens affects histone lysine methylation and histone acetylation in the chick liver, including H3-K36 methylation, H3-K4 methylation, H4-K8 acetylation, and H4-K12 acetylation. Therefore, we speculated that maternal GEN might upregulate PPAR*δ* expression in the chick livers through histone methylation and acetylation. ChIP-qPCR data verified that H3-K36me3 and H4-K12 acetylation were increased at P1 and P2 sites of PPAR*δ* gene promoter in the chick liver after GEN supplementation for LBB hens. H3K36 methylation participates in the regulation of gene transcription. It is reported that large amounts of H3K36me3 are enriched at the 3′-end of the active transcriptional gene in zebrafish somatic cells [65]. In the current experiment, GEN treatment increased the transcriptional levels of H3-K36 methylation-related genes and methyltransferase (ASH 1L, SETD3, NSD1, and SMYD2), as well as SET domain-containing protein 2 (SETD2) in the chick livers. SETD2 is an H3K36 trimethyltransferase that associates with hyperphosphorylated RNA polymerase II and transcriptional elongation [66], which is consistent with the upregulated mRNA transcription from the RNA polymerase II promoter in offspring chicks. NSD1 is a SET domain histone methyltransferase that primarily methylates nucleosomal H3K36. NSD1 depletion can reportedly decrease the levels of H3K36me2 and H3K36me3 [67]. Furthermore, the transcriptional level of Iws1 is higher in the chick liver of the GEN group compared with the CON group. It is reported that Iws1:Spt6:CTD can promote cotranscriptional mRNA synthesis and histone H3K36 methylation regulated by Setd2 [68]. Therefore, GEN supplementation for LBB hens might enhance the epigenetic modification of histone H3-K36 trimethylation at the promoter region of PPAR*δ* in offspring chicks by upregulating Iws1 and methyltransferase expression. Histone methylation, acetylation, and DNA methylation usually have a synergistic effect on gene transcriptional regulation. Transcriptional activation markers such as H3K4me3, H3K36me3, histone acetylation, and hypomethylation often exist in chromatin fragments where gene transcription is active at the same time [69]. The MYST family acts as lysine acetyltransferases, involving in gene expression and DNA damage repair [70]. In this experiment, the different expressed MYST 2 was clustered with H4-K12 acetylation. Thus, GEN supplementation for LBB hens might promote the epigenetic modification of histone H4-K12 acetylation at the promoter region of PPAR*δ* in the chick livers by upregulating MYST 2 expression. Histone methylation and demethylation, as well as acetylation and deacetylation, are dynamically balanced processes. We also found GEN treatment influenced both H3-K4 demethylation and histone monoubiquitination processes in offspring chicks. Considering the ChIP-qPCR data, GEN supplementing for LBB hens might induce H3-K4 methylation or H4-K8 acetylation at other gene sites of offspring chicks.

## 5. Conclusion

Adding GEN into the diet of LBB hens activated the GH-IGFs-PI3K/Akt pathway in offspring chicks, which increased the body weight gain and organ indexes. Maternal GEN supplementation upregulated MnSOD and MT expression in chick livers, which improved the antioxidative capability. Interestingly, GEN treatment activated H3-K36 trimethylation at the promoter of PPARD through Iws1 and methyltransferases, as well as induced H4-K12 acetylation at the promoter region of PPARD through MYST2, which activated the PPAR*δ* signaling pathway in the chick liver. Furthermore, GEN supplementation for LBB hens enhanced fatty acid beta-oxidation, acyl-CoA metabolic process, lipid transport, and cholesterol metabolism in the chick livers. In conclusion, supplementing GEN for LBB hens alters the fatty acid metabolism and growth performance of offspring chicks by epigenetic modification.

## Figures and Tables

**Table 1:** Growth performance of 21-day-old broiler chicks.

Index	CON	GEN	*P* value
Body weight gain (kg)	0.791 ± 0.020	0.824 ± 0.009	0.001
Feed intake (kg)	1.08 ± 0.05	1.11 ± 0.04	0.114
Feed gain ratio	1.37 ± 0.04	1.34 ± 0.04	0.287
Breast muscle rate (%)	21.3 ± 0.87	23.18 ± 1.49	0.031
Abdomen fat rate (%)	0.017 ± 0.001	0.014 ± 0.003	0.094
Heart index (%)	0.52 ± 0.06	0.53 ± 0.29	0.843
Liver index (%)	2.24 ± 0.33	2.73 ± 0.25	0.013

CON: control group, GEN: GEN-treated group. Growth performance indicators, including body weight gain, feed intake, and feed gain ratio, were calculated by the average value of each cage, *n* = 8 cages. The organ indexes include breast muscle and abdominal fat percentage and heart and liver indexes. Organ index = organ weight/body weight. The average value of organ indexes of two chickens in one cage represents the whole replicate, *n* = 8 cages.

**Table 2:** Hepatic antioxidative capability of 21-day-old broiler chicks.

Index	CON	GEN	*P* value
T-AOC (U/mg prot)	0.60 ± 0.21	0.95 ± 0.24	0.014
MDA (mmol/mg prot)	0.94 ± 0.42	0.69 ± 0.16	0.012
CAT (U/mg prot)	14.77 ± 3.05	14.92 ± 5.31	0.949
T-SOD (U/mg prot)	295 ± 48	373 ± 82	0.038
GSH-Px (U/mg prot)	22.81 ± 3.15	25.92 ± 4.37	0.123

CON: control group, GEN: GEN-treated group. T-AOC (total antioxidant capacity); MDA (malondialdehyde); CAT (catalase); T-SOD (total superoxide dismutase); GSH-Px (glutathione peroxidase), *n* = 8.

**Table 3:** The GEN deposition and lipid metabolic indexes of 21-day-old chicks.

Index	CON	GEN	*P* value
Serum^1^			
Cholesterol (mmol/L)	4.16 ± 0.24	3.63 ± 0.20	<0.001
Triglyceride (mmol/L)	0.52 ± 0.05	0.40 ± 0.08	0.043
HDL (mmol/L)	2.24 ± 0.16	2.62 ± 0.17	0.010
LDL (mmol/L)	0.65 ± 0.14	0.53 ± 0.06	0.034

Liver^2^			
GEN (ng/g, d.w)	122.33 ± 11.78	121.57 ± 10.37	0.945
Cholesterol (mmol/g, prot)	0.78 ± 0.07a	0.60 ± 0.09b	0.002
Triglyceride (mmol/g, prot)	0.88 ± 0.12	0.75 ± 0.09	0.063
C12:0 (*μ*mol/g, d.w)	0.07 ± 0.01	0.07 ± 0.01	0.707
C14:0 (*μ*mol/g, d.w)	1.14 ± 0.16	0.61 ± 0.04	0.005
C14:1 (*μ*mol/g, d.w)	0.09 ± 0.01	0.13 ± 0.03	0.071
C15:0 (*μ*mol/g, d.w)	0.05 ± 0.02	0.04 ± 0.01	0.218
C16:0 (*μ*mol/g, d.w)	68.59 ± 13.14	42.53 ± 10.13	0.053
C16:1 (*μ*mol/g, d.w)	6.33 ± 2.20	6.44 ± 1.64	0.947
C17:0 (*μ*mol/g, d.w)	0.15 ± 0.02	0.11 ± 0.03	0.093
C18:0 (*μ*mol/g, d.w)	33.00 ± 7.42	23.30 ± 6.67	0.168
C18:1n9c (*μ*mol/g, d.w)	61.91 ± 8.31	44.58 ± 9.34	0.074
C18:2n6c (*μ*mol/g, d.w)	31.92 ± 8.45	18.78 ± 3.26	0.066
C18:3n6 (*μ*mol/g, d.w)	0.34 ± 0.06	0.39 ± 0.11	0.472
C18:3n3 (*μ*mol/g, d.w)	0.92 ± 0.38	0.62 ± 0.14	0.267
C20:0 (*μ*mol/g, d.w)	0.18 ± 0.01	0.17 ± 0.02	0.534
C20:1 (*μ*mol/g, d.w)	0.39 ± 0.12	0.32 ± 0.11	0.511
C21:0 (*μ*mol/g, d.w)	0.93 ± 0.01	0.76 ± 0.03	0.001
C20:3n6 (*μ*mol/g, d.w)	1.79 ± 0.04	1.53 ± 0.23	0.125
C20:4n6 (*μ*mol/g, d.w)	6.64 ± 0.76	8.30 ± 0.56	0.038
C20:5n3 (*μ*mol/g, d.w)	0.47 ± 0.01	0.40 ± 0.04	0.041
C22:0 (*μ*mol/g, d.w)	0.34 ± 0.08	0.27 ± 0.03	0.256
C22:1n9 (*μ*mol/g, d.w)	0.03 ± 0.01	0.02 ± 0.003	0.354
C23:0 (*μ*mol/g, d.w)	0.04 ± 0.03	0.09 ± 0.01	0.065
C24:0 (*μ*mol/g, d.w)	0.68 ± 0.28	0.49 ± 0.12	0.333
C22:6n3 (*μ*mol/g, d.w)	1.80 ± 0.51	0.85 ± 0.11	0.035
C24:1 (*μ*mol/g, d.w)	0.22 ± 0.08	0.22 ± 0.06	0.992
LCFAs (*μ*mol/g, d.w)	217.60 ± 23.94	150.98 ± 20.73	0.022
n-3 (*μ*mol/g, d.w)	1.39 ± 0.38	1.02 ± 0.19	0.201
n-6 (*μ*mol/g, d.w)	42.69 ± 5.64	27.35 ± 4.62	0.089
SLCFAs (*μ*mol/g, d.w)	105.18 ± 13.81	68.44 ± 16.56	0.042
MUFAs (*μ*mol/g, d.w)	68.97 ± 7.65	51.73 ± 11.04	0.090
PUFAs (*μ*mol/g, d.w)	43.88 ± 8.91	30.88 ± 3.42	0.078
n-6/n-3	31.45 ± 4.07	26.98 ± 0.38	0.131

HDL, LDL (high/low density lipoprotein), LFCAs (long-chain fatty acids); n-3 (n-3 family of LFCAs); n-6 (n-6 family of LFCAs); SLCFAs (saturated LFCAs); MUFAs (monounsaturated LFCAs); PUFAs (polyunsaturated LFCAs); d.w (dry weight); ^1^ *n* = 8; ^2^ *n* = 6.

**Table 4:** The serum hormone level of 21-day-old broiler chicks.

Treatment	T3 (ng/ml)	T4 (ng/ml)	GH (pg/ml)
CON	1.04 ± 0.36	55.81 ± 5.93	1.09 ± 0.09
GEN	1.00 ± 0.20	51.89 ± 5.92	1.19 ± 0.10
*P* value	0.753	0.128	0.067

T3/T4 (triiodothyronine); GH (growth hormone); E2 (estrogen); *n* = 8.

**Table 5:** Results of the PANTHER classification system statistical overrepresentation test of Cuffdiff results (CON vs. GEN).

PANTHER GO-Slim biological process	*Gallus gallus* REFLIST (15782)	Observed	Expected	Over/under	Fold enrichment	*P* value
Metabolic process	4935	1050	822.28	+	1.28	7.99E-17
Lipid metabolic process	544	108	90.64	+	1.19	5.91E-02
Fatty acid metabolic process	151	41	25.16	+	1.63	6.92E-03
Fatty acid biosynthetic process	39	8	6.5	+	1.23	5.34E-01
Unsaturated fatty acid biosynthetic process	6	3	1	+	3	1.26E-01
Fatty acid beta-oxidation	21	8	3.5	+	2.29	3.71E-02
Acyl-CoA metabolic process	27	7	4.5	+	1.56	2.21E-01
Lipid transport	61	17	10.16	+	1.67	3.23E-02
Cholesterol metabolic process	25	6	4.17	+	1.44	4.37E-01
Glycogen metabolic process	30	10	5	+	2	3.68E-02
Cellular ketone body metabolic process	11	3	4.02	+	1.75	6.03E-02
Bile acid metabolic process	2	1	0.06	+	16.99	8.33E-02
Cellular amino acid metabolic process	222	57	36.99	+	1.54	4.41E-03
Developmental growth	446	81	200.37	+	1.51	6.31E-04
Glycolysis	31·	9	5.17	+	1.74	4.69E-02
Negative regulation of apoptotic process	101	31	16.83	+	1.84	3.98E-03
MAPK signaling pathway	297	76	49.49	+	1.54	1.26E-03
Insulin-like growth factor receptor signaling pathway	27	10	51.23	+	1.66	1.21E-03
PPAR signaling pathway	51	14	9.35	+	1.49	2.14E-02
Insulin receptor signaling pathway	82	20	8.68	+	1.79	2.91E-03
I-kappaB kinase/NF-kappaB cascade	41	8	0.83	+	1.17	4.82E-01
Response to interferon-gamma	43	10	7.16	+	1.40	3.26E-01

## Data Availability

The data of growth performance, antioxidative capability, biochemical index, LCFA content, PANTHER statistical overrepresentation test and GO-Slim analysis, relative RNA expression of genes, DEGs clustered in the PPAR signaling pathway, and enrichment level of ChIP-qPCR used to support the findings of this study are included within the article. The data of diet composition, primers, RNA-Seq statistics, different expressed genes, functional cluster analysis of histone modifications, embryonic body sizes, and fragment size after chromatin fragmentation used to support the findings of this study are included within the supplementary information files.

## Abbreviations

CON: Control
ROS: Reactive oxygen species
T3: Triiodothyronine
T4: Tetraiodothyronine
GH: Growth hormone
E2: Estradiol
DEGs: Differentially expressed genes
BWG: Body weight gain
FI: Feed intake
FCR: Feed conversion ratio
GEN: Genistein
ISF: Isoflavone
MT: Metallothionein
T-AOC: Total antioxidant capacity
MDA: Malondialdehyde
CAT: Catalase
T-SOD: Total superoxide dismutase
GSH-Px: Glutathione peroxidase
LBB: Laying broiler breeder
ChIP: Chromatin immunoprecipitation
HDL/LDL: High/low density lipoprotein
LCFAs: Long-chain fatty acids
RPKM: Per kilobase per million
MUFAs: Monounsaturated LFCAs.

## Supplementary Materials

Supplementary 1 A photographic abstract.

Supplementary 2 Assay procedure of chromatin ChIP-qPCR.

Supplementary 3 Table S1: diet composition and nutrient levels.

Supplementary 4 Table S2: primers used for quantitative real-time PCR analysis.

Supplementary 5 Table S3: ChIP-qPCR primer information.

Supplementary 6 Table S4: RNA-Seq statistics of samples used in the experiment.

Supplementary 7 Table 5: the different expressed genes between the CON and GEN groups.

Supplementary 8 Table S6: results of differential expression gene (CON vs. GEN) analysis using Cuffdiff software.

Supplementary 9 Table 7: the functional cluster analysis of histone modifications using DEGs.

Supplementary 10 Figure S1: proportion of the exonic and intronic regions aligned to the reference genome.

Supplementary 11 Figure S2: the effects of maternal GEN on the body sizes of embryos at days 11, 13, 15, 17, and 19.

Supplementary 12 Figure S3: fragment size after chromatin fragmentation.

## References

[1] Cadby C. D., Jones S. M., Wapstra E. (2011). Potentially adaptive effects of maternal nutrition during gestation on offspring phenotype of a viviparous reptile. *Journal of Experimental Biology*.

[2] Gluckman P. D., Cutfield W., Hofman P., Hanson M. A. (2005). The fetal, neonatal, and infant environments—the long-term consequences for disease risk. *Early Human Development*.

[3] Desai M., Hales C. N. (1997). Role of fetal and infant growth in programming metabolism in later life. *Biological Reviews*.

[4] Fu Q., Mcknight R. A., Yu X., Wang L., Callaway C. W., Lane R. H. (2004). Uteroplacental insufficiency induces site-specific changes in histone H3 covalent modifications and affects DNA-histone H3 positioning in *day 0* IUGR rat liver. *Physiological Genomics*.

[5] Gluckman P. D., Hanson M. A., Pinal C. (2005). The developmental origins of adult disease. *Maternal & Child Nutrition*.

[6] Oben J. A., Patel T., Mouralidarane A. (2010). Maternal obesity programmes offspring development of non-alcoholic fatty pancreas disease. *Biochemical and Biophysical Research Communications*.

[7] Shahbazian M. D., Grunstein M. (2007). Functions of site-specific histone acetylation and deacetylation. *Annual Review of Biochemistry*.

[8] Ito K., Barnes P. J., Adcock I. M. (2000). Histone acetylation and deacetylation. *Asthma*.

[9] Greer E. L., Shi Y. (2012). Histone methylation: a dynamic mark in health, disease and inheritance. *Nature Reviews Genetics*.

[10] Hajkova P., Ancelin K., Waldmann T. (2008). Chromatin dynamics during epigenetic reprogramming in the mouse germ line. *Nature*.

[11] Greer E. L., Beese-Sims S. E., Brookes E. (2014). A histone methylation network regulates transgenerational epigenetic memory in *C. elegans*. *Cell Reports*.

[12] Greer E. L., Becker B., Latza C., Antebi A., Shi Y. (2016). Mutation of *C. elegans* demethylase *spr-5* extends transgenerational longevity. *Cell Research*.

[13] Mazur W. M., Duke J. A., Wähälä K., Rasku S., Adlercreutz H. (1998). Isoflavonoids and lignans in legumes: nutritional and health aspects in humans. *The Journal of Nutritional Biochemistry*.

[14] Squadrito F., Marini H., Bitto A. (2013). Genistein in the metabolic syndrome: results of a randomized clinical trial. *The Journal of Clinical Endocrinology & Metabolism*.

[15] Minutoli L., Antonuccio P., Polito F. (2009). Peroxisome proliferator activated receptor *β*/*δ* activation prevents extracellular regulated kinase 1/2 phosphorylation and protects the testis from ischemia and reperfusion injury. *Journal of Urology*.

[16] Tanaka T., Iwasaki S., Asaba H. (2003). 3P-0708 PPAR*δ* agonist ameliorates obesity and insulin resistance through coordinate regulation of fatty acid metabolism in skeletal muscle. *Atherosclerosis*.

[17] Khalaji S., Zaghari M., Ganjkhanloo M., Ghaziani F. (2013). Arginine, soy isoflavone and hydroxypropylmethylcellulose have protective effects against obesity in broiler breeder hens fed on high-energy diets. *British Poultry Science*.

[18] Rüfer C. E., Kulling S. E. (2006). Antioxidant activity of isoflavones and their major metabolites using different in vitro assays. *Journal of Agricultural and Food Chemistry*.

[19] Jiang Z. Y., Jiang S. Q., Lin Y. C., Xi P. B., Yu D. Q., Wu T. X. (2007). Effects of soybean isoflavone on growth performance, meat quality, and antioxidation in male broilers. *Poultry Science*.

[20] Dolinoy D. C., Weidman J. R., Waterland R. A., Jirtle R. L. (2006). Maternal genistein alters coat color and protects *A^vy^* mouse offspring from obesity by modifying the fetal epigenome. *Environmental Health Perspectives*.

[21] Vardi A., Bosviel R., Rabiau N. (2010). Soy phytoestrogens modify DNA methylation of *GSTP1, RASSF1A, EPH2* and *BRCA1* promoter in prostate cancer cells. *In Vivo*.

[22] Lattrich C., Lubig J., Springwald A., Goerse R., Ortmann O., Treeck O. (2011). Additive effects of trastuzumab and genistein on human breast cancer cells. *Anti-Cancer Drugs*.

[23] Li H., Xu W., Huang Y., Huang X., Xu L., Lv Z. (2012). Genistein demethylates the promoter of CHD5 and inhibits neuroblastoma growth in vivo. *International Journal of Molecular Medicine*.

[24] Laker R. C., Wlodek M. E., Connelly J. J., Yan Z. (2013). Epigenetic origins of metabolic disease: the impact of the maternal condition to the offspring epigenome and later health consequences. *Food Science and Human Wellness*.

[25] Saitoh S., Sato T., Harada H., Takita T. (2001). Transfer of soy isoflavone into the egg yolk of chickens. *Bioscience, Biotechnology, and Biochemistry*.

[26] Lv Z., Fan H., Zhang B., Xing K., Guo Y. (2018). Dietary genistein supplementation for breeders and their offspring improves the growth performance and immune function of broilers. *Scientific Reports*.

[27] Ichihara K., Fukubayashi Y. (2010). Preparation of fatty acid methyl esters for gas-liquid chromatography. *Journal of Lipid Research*.

[28] Lv Z., Xing K., Li G., Liu D., Guo Y. (2018). Dietary genistein alleviates lipid metabolism disorder and inflammatory response in laying hens with fatty liver syndrome. *Frontiers in Physiology*.

[29] Fan H., Lv Z., Gan L., Guo Y. (2018). Transcriptomics-related mechanisms of supplementing laying broiler breeder hens with dietary daidzein to improve the immune function and growth performance of offspring. *Journal of Agricultural and Food Chemistry*.

[30] Babraham Bioinformatics FastQC-A quality control tool for high throughput sequence data. http://www.bioinformatics.babraham.ac.uk/projects/fastqc.

[31] FASTX-Toolkit. http://hannonlab.cshl.edu/fastx_toolkit.

[32] Kim D., Pertea G., Trapnell C., Pimentel H., Kelley R., Salzberg S. L. (2013). TopHat2: accurate alignment of transcriptomes in the presence of insertions, deletions and gene fusions. *Genome Biology*.

[33] Oczkowicz M., Świątkiewicz M., Ropka-Molik K., Gurgul A., Żukowski K. (2016). Effects of different sources of fat in the diet of pigs on the liver transcriptome estimated by RNA-seq. *Annals of Animal Science*.

[34] Benjamini Y., Hochberg Y. (1995). Controlling the false discovery rate: a practical and powerful approach to multiple testing. *Journal of the Royal Statistical Society: Series B (Methodological)*.

[35] Saleh A., Alvarez-Venegas R., Avramova Z. (2008). An efficient chromatin immunoprecipitation (ChIP) protocol for studying histone modifications in *Arabidopsis* plants. *Nature Protocols*.

[36] Retana-Márquez S., Juárez-Rojas L., Hernández A. (2016). Comparison of the effects of mesquite pod and *Leucaena* extracts with phytoestrogens on the reproductive physiology and sexual behavior in the male rat. *Physiology & Behavior*.

[37] Shin J. H., Park J. M., Kim J. M., Roh K. S., Jung W. S. (2012). The improvement of laying productivity and egg quality according to providing germinated and fermented soybean for a feed additive. *Korean Journal for Food Science of Animal Resources*.

[38] Rasouli E., Jahanian R. (2015). Improved performance and immunological responses as the result of dietary genistein supplementation of broiler chicks. *Animal*.

[39] Loh B., Maier I., Winar A., Janke O., Tzschentke B. (2004). Prenatal development of epigenetic adaptation processes in poultry: changes in metabolic and neuronal thermoregulatory mechanisms. *Avian and Poultry Biology Reviews*.

[40] Misztal T., Wańkowska M., Górski K., Romanowicz K. (2007). Central estrogen-like effect of genistein on growth hormone secretion in the ewe. *Acta Neurobiologiae Experimentalis*.

[41] Resnicoff M., Sell C., Ambrose D., Baserga R., Rubin R. (1993). Ethanol inhibits the autophosphorylation of the insulin-like growth factor 1 (IGF-1) receptor and IGF-1-mediated proliferation of 3T3 cells. *Journal of Biological Chemistry*.

[42] Zhou J., Kumar T. R., Matzuk M. M., Bondy C. (1997). Insulin-like growth factor I regulates gonadotropin responsiveness in the murine ovary. *Molecular Endocrinology*.

[43] Rosen C. J., Pollak M. (1999). Circulating IGF-I: new perspectives for a new century. *Trends in Endocrinology & Metabolism*.

[44] Twigg S. M., Baxter R. C. (1998). Insulin-like growth factor (IGF)-binding protein 5 forms an alternative ternary complex with IGFs and the acid-labile subunit. *Journal of Biological Chemistry*.

[45] Ma J., Sawai H., Matsuo Y. (2010). IGF-1 mediates PTEN suppression and enhances cell invasion and proliferation via activation of the IGF-1/PI3K/Akt signaling pathway in pancreatic cancer cells. *Journal of Surgical Research*.

[46] Zhu B. Z., Melidou M., Doulias P. T. (2004). Flavonoids protect against hydrogen peroxide-induced cellular DNA damage: the role of iron chelation. *Free Radical Biology & Medicine*.

[47] Finking G., Hanke H., Hess B. (1998). Ansatzpunkte für einen therapeutischen Einsatz von Phyto strogenen (Isoflavonen) bei postmenopausalen Frauen. *Journal für Menopause*.

[48] Borrás C., Gambini J., Gómez-Cabrera M. C. (2006). Genistein, a soy isoflavone, up-regulates expression of antioxidant genes: involvement of estrogen receptors, ERK1/2, and NF*κ*B. *The FASEB Journal*.

[49] Sato M., Bremner I. (1993). Oxygen free radicals and metallothionein. *Free Radical Biology & Medicine*.

[50] Kuo S. M., Leavitt P. S., Lin C. P. (1998). Dietary flavonoids interact with trace metals and affect metallothionein level in human intestinal cells. *Biological Trace Element Research*.

[51] Lau C., Rogers J. M., Desai M., Ross M. G. (2011). Fetal programming of adult disease: implications for prenatal care. *Obstetrics & Gynecology*.

[52] Zierau O., Power K. A. (2011). Lifelong and prenatal effects of phytoestrogens. *Current Bioactive Compounds*.

[53] Kim S., Lee K.-H., Hong I. (2006). Genistein stimulates differentiation in epidermal keratinocyte through activation of PPARs. *The FASEB Journal*.

[54] Lee K. Y., Schulz H. (1979). Isolation, properties, and regulation of a mitochondrial acyl coenzyme A thioesterase from pig heart. *Journal of Biological Chemistry*.

[55] Hunt M. C., Alexson S. E. H. (2002). The role acyl-CoA thioesterases play in mediating intracellular lipid metabolism. *Progress in Lipid Research*.

[56] Danesch U., Weber P. C., Sellmayer A. (1994). Arachidonic acid increases c-*fos* and *Egr-1* mRNA in 3T3 fibroblasts by formation of prostaglandin E_2_ and activation of protein kinase C. *Journal of Biological Chemistry*.

[57] Gordon S. M., Hofmann S., Askew D. S., Davidson W. S. (2011). High density lipoprotein: it’s not just about lipid transport anymore. *Trends in Endocrinology & Metabolism*.

[58] Bhathena S. J., Velasquez M. T. (2002). Beneficial role of dietary phytoestrogens in obesity and diabetes. *The American Journal of Clinical Nutrition*.

[59] Dang Z. C., Audinot V., Papapoulos S. E., Boutin J. A., Löwik C. W. G. M. (2003). Peroxisome proliferator-activated receptor *γ* (PPAR*γ*) as a molecular target for the soy phytoestrogen genistein. *Journal of Biological Chemistry*.

[60] Wang Y. X., Lee C. H., Tiep S. (2003). Peroxisome-proliferator-activated receptor *δ* activates fat metabolism to prevent obesity. *Cell*.

[61] Libertini L. J., Smith S. (1978). Purification and properties of a thioesterase from lactating rat mammary gland which modifies the product specificity of fatty acid synthetase. *Journal of Biological Chemistry*.

[62] Holst D., Grimaldi P. A. (2002). New factors in the regulation of adipose differentiation and metabolism. *Current Opinion in Lipidology*.

[63] Herceg Z. (2007). Epigenetics and cancer: towards an evaluation of the impact of environmental and dietary factors. *Mutagenesis*.

[64] Holliday R. (1990). Mechanisms for the control of gene activity during development. *Biological Reviews of the Cambridge Philosophical Society*.

[65] Wu S., Zhang H., Bradley C. (2011). Genes for embryo development are packaged in blocks of multivalent chromatin in zebrafish sperm. *Genome Research*.

[66] Xie P., Tian C., An L. (2008). Histone methyltransferase protein SETD2 interacts with p53 and selectively regulates its downstream genes. *Cellular Signalling*.

[67] Qiao Q., Li Y., Chen Z., Wang M., Reinberg D., Xu R. M. (2011). The structure of NSD1 reveals an autoregulatory mechanism underlying histone H3K36 methylation. *Journal of Biological Chemistry*.

[68] Yoh S. M., Lucas J. S., Jones K. A. (2008). The Iws1:Spt6:CTD complex controls cotranscriptional mRNA biosynthesis and HYPB/Setd2-mediated histone H3K36 methylation. *Genes & Development*.

[69] Handy D. E., Castro R., Loscalzo J. (2011). Epigenetic modifications. *Circulation*.

[70] Vonlaufen N., Naguleswaran A., Coppens I., Sullivan W. J. (2010). MYST family lysine acetyltransferase facilitates ataxia telangiectasia mutated (ATM) inase-mediated DNA damage response in *Toxoplasma gondii*. *Journal of Biological Chemistry*.

