# Supplementary material for: Supplementing Genistein for Breeder Hens Alters the Fatty Acid Metabolism and Growth Performance of Offsprings by Epigenetic Modification
Source: Oxid Med Cell Longev. 2019 Mar 26;2019:9214209. doi: 10.1155/2019/9214209 (PMC6458848; doi:10.1155/2019/9214209)
Supplement: Supplementary 1 — A photographic abstract. [file 9214209.f1.docx]

**
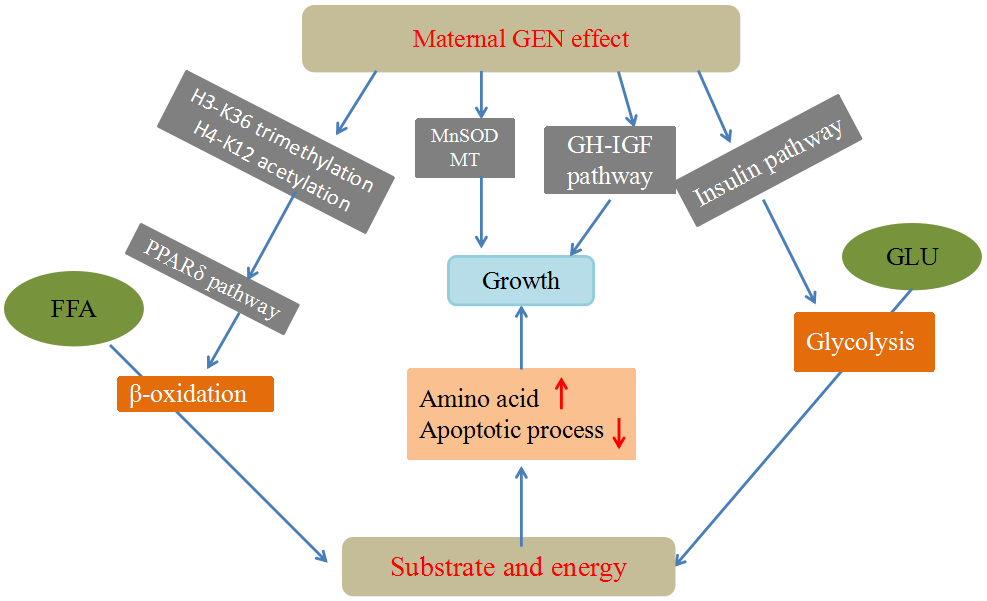
**

**A photographic abstract:**

Adding GEN into the diet of LBB hens activated the GH-IGFs-PI3K/Akt pathway in offspring, which increased body weight gain and organ indexes. Maternal GEN supplementation up-regulated MnSOD and MT expression in offspring livers, which improved the antioxidative capability. Interestingly, GEN supplementation activated H3-K36 trimethylation at the promoter of PPARD methyltransferases and induced H4-K12 acetylation at the promoter region of PPARD, which up-regulated PPARδ expression in offspring liver. Additionally, GEN supplementation influenced, [fatty acid beta-oxidation](http://www.pantherdb.org/panther/category.do?categoryAcc=GO:0006635), which decreased LCFA and cholesterol levels in chick livers. Therefore, maternal GEN supplementation can regulate offspring metabolism and development through activating IGF and PPAR signalling pathways in offspring.
