# Supplementary material for: Supplementing Genistein for Breeder Hens Alters the Fatty Acid Metabolism and Growth Performance of Offsprings by Epigenetic Modification
Source: Oxid Med Cell Longev. 2019 Mar 26;2019:9214209. doi: 10.1155/2019/9214209 (PMC6458848; doi:10.1155/2019/9214209)
Supplement: Supplementary 2 — Assay procedure of chromatin ChIP-qPCR. [file 9214209.f2.docx]

**Assay procedure of chromatin CHIP-qPCR**

Section A. Chromatin Crosslink with Tissue Samples

1. For each sample, choose 100-400mg fresh or frozen tissue sample. Cut the tissue into small pieces (approximately 1mm cubes) using a razor blade.

2. Transfer tissue pieces into a tube, place the tube on ice and homogenize the contents by tissue homogenizer.

3. Pellet the cells from step 2 by centrifugation for 5 minutes at 1000 x g at 4 degree.

4. Remove the supernatant and discard. Resuspend the pellet in 10ml prior ice-cold PBS.

5. Add 275μl 37% formaldehyde to cells from step 4 and mix thoroughly, leave at room temperature, incubate on a shaking platform for 10 minutes.

6. Stop the crosslink reaction by adding 500μl glycine solution(10x glycine solution),

incubate at room temperature for 5 minutes on a shaking platform.

7. Pellet the cells from step 6 by centrifugation for 5 minutes at 1000 x g at 4 degree.

8. Wash cells with 1ml ice-cold 1 x PBS, spin down at 1000 x g for 5 minutes at 4 degree, then discard supernatant.

9. Aspirate PBS, flash freeze and keep the cell pellet frozen at -80 degree until needed.

Note:

In standards protocol, chromatin crosslink for 10 minute by 1% concentration

formaldehyde prior shearing while some protein/DNA combinations may work better

with shorter fixation time.

Section B. Chromatin Fragmentation

1. Add 200μl cell Lysis Buffer containing 1μl protease inhibitor (1000x Protease

Inhibitor Cocktail), Incubate for 30 minutes on a rocker at 4 degree.

2. Add 800μl ChIP Dilution Buffer to final volume 1ml.

3. Chromatin shearing by microtip probe sonicator:

a) Amplitude 30-50%

b) Process time 10-20 minutes

c) Pulse on time 0.5s

d) Pulse off time 0.5s

4. After sonication, centrifuge at 10000 x g for 15 minutes at 4 degree. Transfer the

supernatant to a new tube.

5. Transfer 50μl supernatant as input, add 50μl Dilution Buffer and 5μl Proteinase K,

incubate at 65 degree for 4 hours or overnight to reverse crosslink.

6. Purify reverse corsslinked DNA using DNA purification kit, elute by 50μl Elution

Buffer .

7. Load 5μl input DNA on a 2% agarose gel to check chromatin shearing performance.

8. Fragmented chromatin could be store at -80 degree until needed.

Note:

For different cell types and stimulation conditions, chromatin shearing programs may be different. Prior ChIP assay, chromatin shearing conditions should be optimized.

Section E. Reverse Corsslink and DNA Purification

1. Wash beads by adding 1ml ice-cold Wash buffer I, incubate at 4 degree on a rocker for 5 minutes.

2. Place tube on a magnetic particle collector for 2 minute then discard supernatent.

3. Wash beads by adding 1ml ice-cold Wash buffer II, incubate at 4 degree on a rocker

for 5 minutes.

4. Place tube on a magnetic particle collector for 2 minute then discard supernatant.

5.Wash beads by adding 1ml ice-cold Wash buffer III, incubate at 4 degree on a rocker

for 5 minutes.

6. Place tube on a magnetic particle collector for 2 minute then discard supernatant.

7. Wash beads by adding 1ml ice-cold TE buffer, incubate at 4 degree on a rocker for 5 minutes.

8. Place tube on a magnetic particle collector for 2 minute then discard supernatant.

9. Add 200μl ChIP Elution Buffer and 10μl Proteinase K, incubate at 65 degree for 4

hours or overnight to reverse crosslink.

10. After reverse crosslink, place tube on magnetic particle collector for 2 minutes then transfer supernatant to a new tube.

11. Purify reverse corsslinked DNA, elute by 60-100μl Elution Buffer.

12. ChIP DNA could be used for ChIP-qPCR detection or store at -80 degree until used.
