# Supplementary material for: Supplementing Genistein for Breeder Hens Alters the Fatty Acid Metabolism and Growth Performance of Offsprings by Epigenetic Modification
Source: Oxid Med Cell Longev. 2019 Mar 26;2019:9214209. doi: 10.1155/2019/9214209 (PMC6458848; doi:10.1155/2019/9214209)
Supplement: Supplementary 3 — Table S1: diet composition and nutrient levels. [file 9214209.f3.docx]

**Supplementary Table 1**. **Diet composition and nutrient levels**

| Ingredient | Corn-miscellaneous meal diet (%) | Feed formula (0-3 weeks, %) |
| --- | --- | --- |
| Corn | 68.99 | 53.28 |
| Soybean meal | 4 | 38.57 |
| Corn protein | 9.15 | — |
| De-gossypol cottonseed protein | 6 | — |
| Limestone | 7.76 | 1.05 |
| Soybean oil | 0.5 | 3.7 |
| Dicalcium phosphate | 2.09 | 1.98 |
| NaCl | 0.35 | 0.35 |
| ^a^Trace mineral Premix | 0.3 | 0.3 |
| Choline chloride (50%) | 0.12 | 0.3 |
| Mycotoxin adsorbent | 0.1 | — |
| DL- methionine | 0.0515 | 0.22 |
| ^b^Vitamin premix | 0.035 | 0.02 |
| Santoquin | 0.03 | 0.03 |
| Phytase | 0.016 | — |
| 4% Flavomycin | 0.015 | — |
| Lysine•HCl (8%) | 0.373 | 0.12 |
| Threonine | 0.0664 | — |
| Tryptophan | 0.0481 | — |
| Tatal | 100 | 100 |
| Avian metabolic energy MC/kg | 2.83 | 2.9526 |
| Crude protein | 16.1 | 21.6 |
| Calcium | 3.48 | 1.051 |
| Tatal phosphorus | 0.678 | 0.7 |
| Available phosphorus | 0.47 | 0.45 |
| Methionine | 0.34 | 0.5 |
| Lysine | 0.805 | 1.15 |
| Met+Cys | 0.626 | 0.86 |
| Threonine | 0.6 | 0.8 |
| Tryptophan | 0.18 | — |

^a^The following was supplied per kg complete diet: Cu, 8 mg; Zn, 75 mg; Fe, 80 mg; Mn, 100 mg; Se, 0.15 mg; I, 0.35 mg.
^b^The following was supplied per kg complete diet: vitamin A, 12500 IU; vitamin D3, 2500 IU; vitamin E, 30 IU; vitamin K3, 2.65 mg; thiamine, 2 mg; riboflavin, 6 mg; vitamin B12, 0.025 mg; biotin, 0.0325 mg; folic acid, 1.25 mg; pantothenic acid, 12 mg; niacin, 50 mg.
