# Supplementary material for: Supplementing Genistein for Breeder Hens Alters the Fatty Acid Metabolism and Growth Performance of Offsprings by Epigenetic Modification
Source: Oxid Med Cell Longev. 2019 Mar 26;2019:9214209. doi: 10.1155/2019/9214209 (PMC6458848; doi:10.1155/2019/9214209)
Supplement: Supplementary 4 — Table S2: primers used for quantitative real-time PCR analysis. [file 9214209.f4.docx]

**Supplementary Table 2. Primers used for quantitative real-time PCR analysis**

| Gen name | 5' primer | Prod side |
| --- | --- | --- |
| SOD3 | F: TTGTGATCCATGAGCAGGAA | 122 |
|  | R: TTGTTGCAGATCCCAATCAC |  |
| HADHA | F:TGTCTCCACCTCCACTGCTCTTC | 80 |
|  | R:TGAAGCGGACAACAGCAACATCTC |  |
| ECHS1 | F:AGGAGCTGAGACGCGCACTG | 149 |
|  | R:GAAGCCGCCAGCATAGCACTC |  |
| ESR1 | F: TAGAGGGCATGGTGGAAATC | 122 |
|  | R: CACACCAGAATTGAGCAGGA |  |
| HMGCL | F: TGAGTGTGGTTGATGCTTCTGTCG | 152 |
|  | R: GCCGGTGTCCATGAGCTTCTG |  |
| PPARδ | F:TGAATGACCAAGTGACTCTGCTGAAG | 320 |
|  | R: CAGTGCTCGGAGGATGTTGTCTTG |  |
| ACOT9 | F: CAGTAACACCGAGATGGAGACAAGG | 289 |
|  | R: GCTGTTCTTGAAGGCGTTGTTGAC |  |
| ECI1 | F: GCTGCCTCCTCTCCCTCA | 349 |
|  | R: GGAACAGCCAGCCACTTG |  |
| ACOX2 | F: GACAGAGTTGGGACATGGGACATATCTT | 114 |
|  | R: GCGTGGGGCTGTGTATCACAA A |  |
| HMGCS2 | F: TCTGCTCTGACCGAGAGGAT | 117 |
|  | R: TTGTCTCCGTTCCAACTTCC |  |
| ACAD8 | F: GATTGTCACGTCTCGATACCTCCATC | 137 |
|  | R: CCTGTGCCTCTGTTCCTCATTGC |  |
| GAPDH | F:TGCTGCCCAGAACATCATCC | 120 |
|  | R:ACGGCAGGTCAGGTCAACAA |  |
| CYP7A1 | F:GAGCTTGAGGCACGAGAACC | 119 |
|  | R:GGTGTGTCTTGGCCTTCTCC |  |
| MT1 | F:ACGTGTGGAGACAACTGCAA | 96 |
|  | R:TGCACACTTGGCACATCCT |  |
| IGF1 | F: TGTACTGTGCTCCAATAAAGC | 127 |
|  | R: CTGTTTCCCCACAGCCTTA |  |
| SLC27A1 | F: TACGGAGCCACCGAGTGCAACT | 160 |
|  | R: CGCACAGCCCTCTGGAATCACG |  |

*F represent forward, R represent reward
