# Supplementary material for: Supplementing Genistein for Breeder Hens Alters the Fatty Acid Metabolism and Growth Performance of Offsprings by Epigenetic Modification
Source: Oxid Med Cell Longev. 2019 Mar 26;2019:9214209. doi: 10.1155/2019/9214209 (PMC6458848; doi:10.1155/2019/9214209)
Supplement: Supplementary 5 — Table S3: ChIP-qPCR primer information. [file 9214209.f5.docx]

**Supplementary Table 3. ChIP-qPCR primer information**

|  | Forward | Reverse | PCR size |
| --- | --- | --- | --- |
| P1 | GGTAAAGAGGGAAGGGTGGA | CCCTGTCTGAGACCTTGAGC | 137 bp |
| P2 | AGTGACTACCGCGTCACCA | GGAAGGAGGTGTGCGTCAT | 102 bp |
| Location | | | |
| P1 | chr26:4048020+4048156 | | |
| P2 | chr26:4047862+4047963 | | |
