# Supplementary material for: Supplementing Genistein for Breeder Hens Alters the Fatty Acid Metabolism and Growth Performance of Offsprings by Epigenetic Modification
Source: Oxid Med Cell Longev. 2019 Mar 26;2019:9214209. doi: 10.1155/2019/9214209 (PMC6458848; doi:10.1155/2019/9214209)
Supplement: Supplementary 6 — Table S4: RNA-Seq statistics of samples used in the experiment. [file 9214209.f6.docx]

**Supplementary Table 4. RNA-Seq statistics of samples used in the experiment**

| Sample ID | Raw reads | Q30 value (%) | Mean quality score (PF) | Clean reads | Read1 mapped reads | Read2 mapped reads | Mapping ratio (%)^a^ |
| --- | --- | --- | --- | --- | --- | --- | --- |
| CON1 | 87620830 | 91.13 | 35.81 | 82733822 | 34297589 | 34190836 | 82.8 |
| CON2 | 86830340 | 90.97 | 35.78 | 81773986 | 35295956 | 35198297 | 82.1 |
| CON3 | 78145526 | 91.35 | 35.84 | 73869122 | 30868806 | 30868806 | 83.6 |
| CON4 | 92007936 | 91.2 | 35.81 | 87132554 | 36427631 | 36427631 | 85.1 |
| GEN1 | 79757656 | 90.8 | 35.72 | 75440666 | 34183206 | 31828357 | 84.5 |
| GEN2 | 88405862 | 91.63 | 35.9 | 83685086 | 35410936 | 35410936 | 84.6 |
| GEN3 | 82931990 | 91.34 | 35.82 | 78478360 | 32895062 | 32895062 | 83.8 |
| GEN4 | 83513140 | 91.43 | 35.85 | 78975760 | 33053311 | 32973655 | 83.5 |

CON the control group; GEN the GEN-treated group. ^a^Mapping ratio = mapped reads/all reads.
