# Supplementary material for: Supplementing Genistein for Breeder Hens Alters the Fatty Acid Metabolism and Growth Performance of Offsprings by Epigenetic Modification
Source: Oxid Med Cell Longev. 2019 Mar 26;2019:9214209. doi: 10.1155/2019/9214209 (PMC6458848; doi:10.1155/2019/9214209)
Supplement: Supplementary 8 — Table S6: results of differential expression gene (CON vs. GEN) analysis using Cuffdiff software. [file 9214209.f8.docx]

**Supplementary Table 6. Results of differential expression gene (CON vs GEN) analysis using Cuffdiff software**

| Gene | Value_CON | Value_GEN | Log2 (fold change) | P_value | Q_value |
| --- | --- | --- | --- | --- | --- |
| ACSM3 | 19.25 | 32.18 | 0.74 | 0.0003 | 0.0018 |
| PMVK | 34.4 | 77.34 | 1.17 | 0.0001 | 0.0005 |
| ACOT9 | 5.18 | 9.37 | 0.85 | 0.0005 | 0.0028 |
| HMGCS2 | 567.83 | 920.71 | 0.7 | 0.0052 | 0.0166 |
| ACADL | 339.21 | 531.46 | 0.65 | 0.011 | 0.0284 |
| ECHDC1 | 7.53 | 11.5 | 0.61 | 0.0079 | 0.0226 |
| ECI1 | 117.69 | 259.26 | 1.14 | 0.0001 | 0.0005 |
| ACOX2 | 80.38 | 130.25 | 0.7 | 0.0005 | 0.0031 |
| ACAT2 | 236.19 | 362.59 | 0.62 | 0.0064 | 0.0194 |
| HADHA | 106.79 | 194.01 | 0.86 | 0.0001 | 0.0005 |
| PPARD | 3.21 | 7.33 | 1.19 | 0.0001 | 0.0005 |
| SLC27A1 | 4.96 | 7.81 | 0.65 | 0.0034 | 0.0122 |
| APOA5 | 213.7 | 355.11 | 0.73 | 0.0004 | 0.0024 |
| PLTP | 2.19 | 5.31 | 1.28 | 0.0003 | 0.0021 |
| ACSL5 | 96.26 | 173.67 | 0.85 | 0.0001 | 0.0005 |
| FABP3 | 2.19 | 5.69 | 1.38 | 0.0032 | 0.0116 |
| CYP7A1 | 24.27 | 71.43 | 1.56 | 0.0001 | 0.0005 |
| ACADL | 339.21 | 531.46 | 0.65 | 0.011 | 0.0284 |
| VLDL | 2.23 | 4.89 | 1.13 | 0.0001 | 0.0005 |
| SOD3 | 5.07 | 20.92 | 2.05 | 0.0001 | 0.0005 |
| IGF-1 | 13.19 | 19.8 | 0.59 | 0.001 | 0.0049 |
| IGFBP1 | 4.85 | 7.7 | 0.67 | 0.0031 | 0.0114 |
| SETD3 | 2.49 | 3.88 | 0.64 | 0.0038 | 0.0133 |
| SETD2 | 11.33 | 15.97 | 0.49 | 0.0102 | 0.027 |
| IWS1 | 18.75 | 37.1 | 0.98 | 0.0001 | 0.0005 |
| MYST2 | 5.17 | 8.48 | 0.71 | 0.0002 | 0.0015 |

Fold change= value_GEN/value_CON; q_value = false discovery rate (FDR)
