# Supplementary material for: Supplementing Genistein for Breeder Hens Alters the Fatty Acid Metabolism and Growth Performance of Offsprings by Epigenetic Modification
Source: Oxid Med Cell Longev. 2019 Mar 26;2019:9214209. doi: 10.1155/2019/9214209 (PMC6458848; doi:10.1155/2019/9214209)
Supplement: Supplementary 10 — Figure S1: proportion of the exonic and intronic regions aligned to the reference genome. [file 9214209.f10.doc]

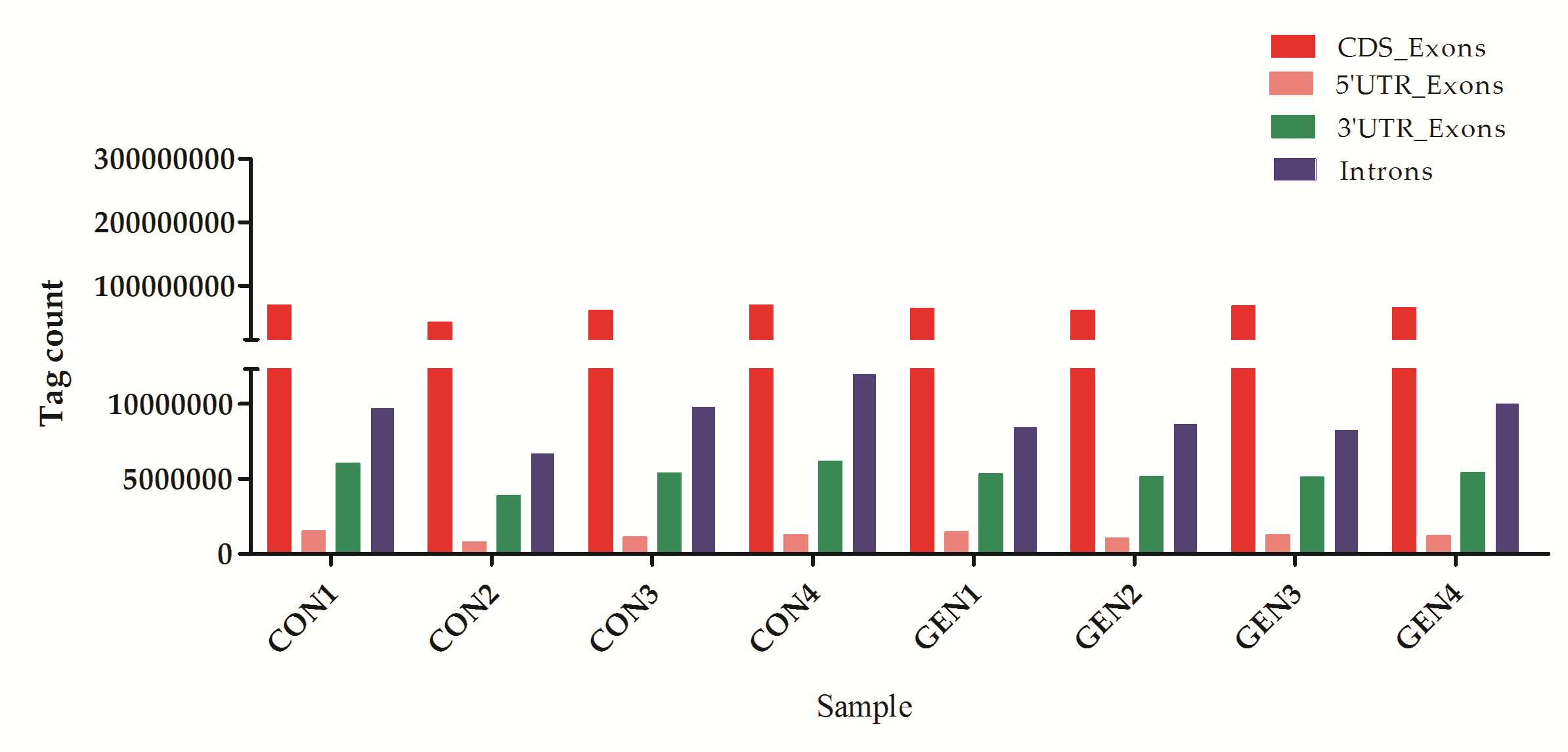


**Supplementary Figure 1.**Proportion of exonic and intronic regions aligned to the reference genome. CON the control group; GEN the GEN-treated group. Tag count is the number of reads mapped to the exon and intron regions of the Gallus gallus genome.
