# Supplementary material for: Supplementing Genistein for Breeder Hens Alters the Fatty Acid Metabolism and Growth Performance of Offsprings by Epigenetic Modification
Source: Oxid Med Cell Longev. 2019 Mar 26;2019:9214209. doi: 10.1155/2019/9214209 (PMC6458848; doi:10.1155/2019/9214209)
Supplement: Supplementary 11 — Figure S2: the effects of maternal GEN on the body sizes of embryos at days 11, 13, 15, 17, and 19. [file 9214209.f11.docx]

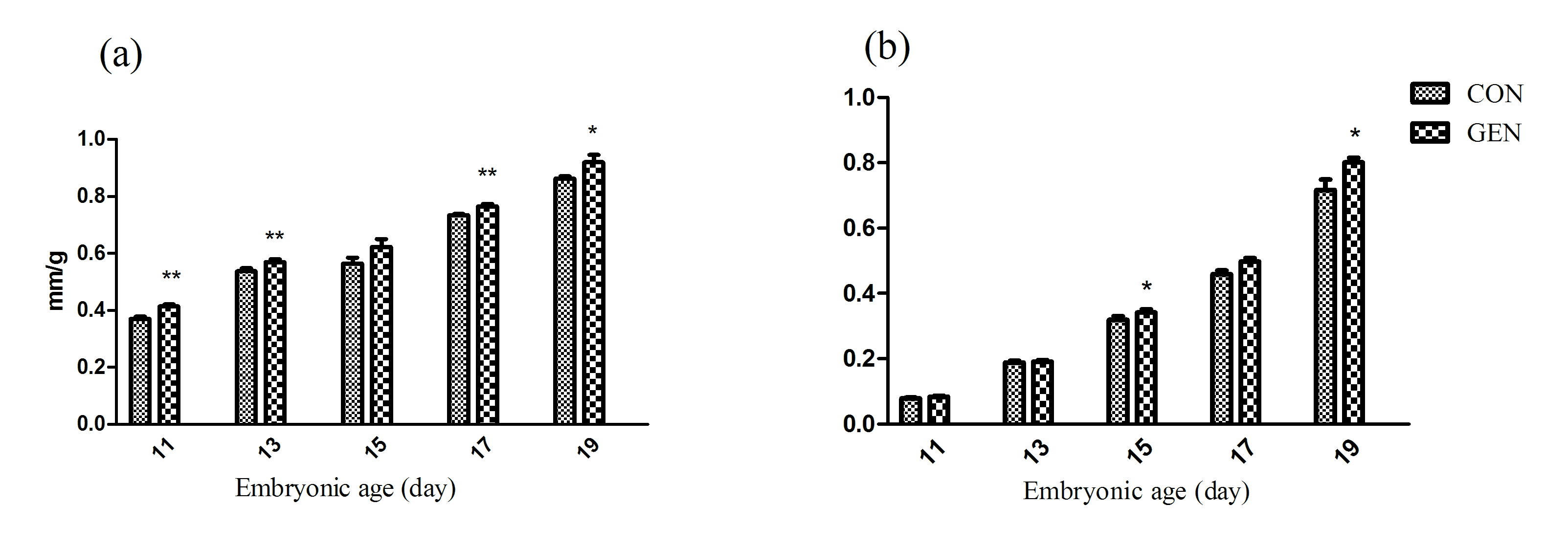


**Supplementary Figure 2.** The effects of maternal GEN on the body sizes of embryos at days 11, 13, 15, 17 and 19 (n=8, Mean±SD). (a) Relative embryo length (body length/egg weight, mm/g); (b) relative embryo weight (wet body weight/egg weight). **presents P<0.05; * presents P<0.10.
