# Supplementary material for: Supplementing Genistein for Breeder Hens Alters the Fatty Acid Metabolism and Growth Performance of Offsprings by Epigenetic Modification
Source: Oxid Med Cell Longev. 2019 Mar 26;2019:9214209. doi: 10.1155/2019/9214209 (PMC6458848; doi:10.1155/2019/9214209)
Supplement: Supplementary 12 — Figure S3: fragment size after chromatin fragmentation. [file 9214209.f12.docx]

**
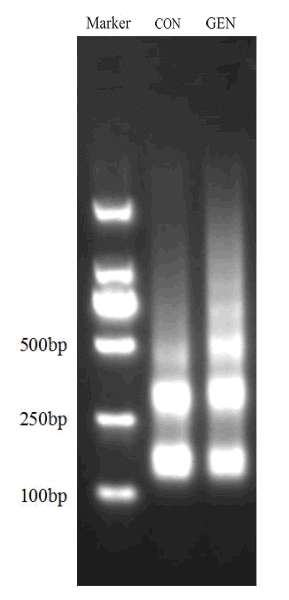
**

**Supplementary Figure 3.** Fragment size after chromatin fragmentation. CON, the control group sample; GEN, the GEN-treated group sample.
